# A new effect of intravenous iron treatment in pregnancy: contraction in nonstress test and timing of labor

**DOI:** 10.1590/1806-9282.20231608

**Published:** 2024-07-19

**Authors:** Mohammad İbrahim Halilzade, İnci Halilzade, Mahmut Kuntay Kokanalı

**Affiliations:** 1 University of Health Sciences, Ankara City Hospital, Department of Obstetrics and Gynecology – Ankara, Turkey.

**Keywords:** Pregnancy, Iron, Calcium, Anemia, Iron-deficiency, Cesarean section, Fetal monitoring

## Abstract

**OBJECTIVE:**

The aim of this study was to elucidate the cause and results of contractions occurring in term pregnant women receiving intravenous iron therapy.

**METHODS:**

During 2019–2020, 136 pregnant women beyond 35 weeks of gestation, who received intravenous iron treatment due to iron deficiency anemia, were included through retrospective screening. Iron deficiency anemia was defined as having hemoglobin levels <10 g/dL and ferritin levels <15 ng/mL, and the pregnant women underwent nonstress test before and after treatment.

**RESULTS:**

The average treatment week for the pregnant women was 36.82±0.74, and the presence of regular contractions in post-treatment follow-up nonstress tests was 72.1% (n=98). The average week of birth was 38.48±1.60. Pregnant women with contractions who had previous cesarean were found to have a mean delivery week of 36.82±0.67, which was statistically significant earlier than for nulliparous and multiparous women (p<0.001).

**Conclusion:**

In pregnant women with iron deficiency anemia who were beyond 35 weeks, temporary regular contractions may be observed in the nonstress test following intravenous iron replacement. We think that this effect may lead to early term birth in pregnant women with a history of cesarean section. It needs to be confirmed by further prospective studies and animal studies.

## INTRODUCTION

Iron deficiency anemia during pregnancy is a global public health problem, affecting approximately 56% of pregnant women in developing countries^1^. Since iron deficiency anemia is associated with adverse maternal and neonatal outcomes during pregnancy, its treatment is crucial^1^. Among these adverse effects are first-trimester pregnancy losses, placental abruption, preterm birth, postpartum hemorrhage, hysterectomy, fetal malformation, growth restriction, stillbirth, and maternal death^2,3^.

According to the World Health Organization guidelines, the criteria for anemia during pregnancy are defined as hemoglobin (Hb) levels <11 g/dL in the first and third trimesters, and <10.5 g/dL for the second trimester^4^. In cases where there is non-compliance with oral treatment or when anemia persists despite oral treatment, intravenous (IV) iron therapy is considered a good alternative option^5^. In addition, the importance of IV iron preparations in the rapid and effective treatment of prenatal anemia in the third trimester has been demonstrated. The recent UK guidelines especially recommend considering IV iron supplementation for anemia treatment after 34 weeks of pregnancy^6^.

Iron (Fe) is an element that is absorbed intestinally through divalent metal transporter 1 (DMT1) receptors^7^. It has been demonstrated that high levels of calcium (Ca) competitively reduce the absorption of iron by competing with iron at the receptor level^8^. Additionally, some studies have shown that this effect is short-lived and does not significantly impact iron absorption^7,9^. However, it has not yet been clearly established whether calcium absorption is affected in cases of high iron concentration.

In our clinic, we applied pre- and post-treatment nonstress test (NST) to pregnant women beyond 35 weeks who require IV iron therapy to monitor fetal well-being. We observed that in the majority of our patients there were no uterine contractions before the treatment, but regular uterine contractions occurred afterwards. The aim of this study was to elucidate the cause of this situation and to understand whether these contractions occurring in term pregnant women receiving IV iron therapy lead to birth before the expected delivery date.

## METHODS

As part of the research, a total of 159 pregnant women beyond 35 weeks, who received IV iron therapy due to iron deficiency anemia, participated in the study conducted at our hospital between 2019 and 2020. Local ethics committee approval was obtained from the same hospital (Ankara Bilkent City Hospital Ethics Committee No. 2, Approval Date: 2021, Approval No: 21/1029). This is a retrospective cohort study.

Pregnant women who had a singleton pregnancy between 35 and 37 weeks of gestation and received IV iron treatment due to existing anemia (despite oral iron therapy) according to WHO criteria were included in our study. Iron deficiency anemia was diagnosed when the hemoglobin was <10 g/dL and ferritin was <15 ng/mL^10^. Women who had any complications in pregnancy (preeclampsia, diabetes mellitus, IUGR, malpresentation, trauma, etc.), who had contractions detected on NST prior to IV iron treatment, and who had insufficient data were excluded from the study. IV ferric carboxymaltose treatment was given to the pregnant women. The reason for this was based on the effectiveness and safety of IV ferric carboxymaltose infusion in the treatment of iron deficiency anemia during pregnancy^11^. IV iron doses required to correct iron deficiency anemia when Hb was <10 g/dL according to body weight were 500 mg (<35 kg), 1,500 mg (>35 to<70 kg), and 2,000 mg (>70 kg). The maximum recommended iron infusion dose was 1,000 mg of iron/week (1,000 mg in the first session and the remaining amount in the second session after 1 week)^10^. Ferric carboxymaltose was administered as a single dose of 1,000 mg via IV drip infusion, to be completed in 15 min.

All pregnant women scheduled to receive IV iron therapy underwent pre-treatment NST. Due to the reported adverse effects of IV iron therapy^12^, patients were subjected to post-treatment NST, and they were kept under observation for 2 h. The patients’ files were reviewed, and NST records prior to and after IV iron therapy were collected. The contraction status during NST, gravida, parity, the hospitalization duration, previous mode of delivery, gestational week at which they received IV iron therapy, the gestational week of delivery, mode of delivery, and the need for neonatal intensive care were determined for these patients. Regular contractions in the NST were defined as the presence of at least four contractions lasting 40 s or longer within 20 min.

The sample size estimation was performed by using G*Power version 3.0.10 (Franz Faul, Universitat Kiel, Kiel, Germany). With a Cohen's effect size of 0.5, alpha error of 0.05, and power of 80%, the minimum number of participants required was calculated as 128. Statistical analyses were performed using SPSS version 22 (IBM Corp., Armonk, NY, USA). The normal distribution of data was examined using the Shapiro-Wilk test. Continuous and normal distributed variables were presented as mean±standard deviation, and within-group differences were evaluated using Student's t-test. Continuous and non-normal distributed variables were presented as median (minimum–maximum), and the differences between variables were analyzed using the Mann-Whitney U-test. Categorical variables were presented as numbers (percentages), and differences between categorical data were compared using the chi-square test or Fisher's exact test. p-Values below 0.05 were considered statistically significant.

## RESULTS

Out of the 159 patients who were selected to receive IV treatment due to iron deficiency anemia, 23 were excluded from the study because they had contractions during the pre-treatment NSTs, 8 due to lack of data, and 2 due to complications, resulting in a total of 136 participants. The characteristics of the pregnant women included in the study are shown in Table 1. The average gestational week for the treatment was determined to be 36.82±0.74. In the post-treatment follow-up NST, the presence of regular contractions was observed in 72.1% (n=98) of the participants (Table 1).

**Table 1:** Characteristics of the pregnant women included in the study.

Characteristics	n=136
Age	27.64±5.52
Hemoglobin (g/dL)	8.78±0.66
Ferritin (ng/mL)	4.34±2.78
Gravida	2 (1–7)
Parity	1 (0–6)
Nulliparity	38 (27.9)
Pre-existing cesarean delivery	56 (41.2)
Treatment week	36.82±0.74
Presence of post-treatment contraction	98 (72.1)
Delivery week	38.48±1.60
Post-treatment cesarean delivery	59 (43.4)

The data are presented as mean ± standard deviation, median (minimum-maximum), and number (%).

The delivery weeks based on the presence of contractions in different conditions are shown in Table 2. Pregnant women who had contractions in the NST after IV iron therapy were found to have significantly earlier delivery weeks (p<0.001). In cases with contractions, the delivery week for women who had previous cesarean deliveries was determined as 36.82±0.67, which was statistically significant earlier than for nulliparous and multiparous pregnant women (p<0.001) (Table 3). This statistical significance is attributed to the cesarean group, and no statistically significant difference was found between nulliparous and multiparous pregnant women.

**Table 2:** The delivery weeks based on the presence of contractions in different conditions.

	Presence of contractions after treatment		p-value
	Contraction (+) n=98	Contraction (-) n=38	
Delivery week	38.03±1.56	39.64±1.00	**<0.001**
	**Presence of contractions in nulliparous pregnant women**		
	**Contraction (+) n=20**	**Contraction (-) n=18**	
Delivery week	39.51±1.25	39.82±0.94	0.395
	**Presence of contractions in multiparous pregnant women**		
	**Contraction (+) n=32**	**Contraction (-) n=10**	
Delivery week	38.83±1.32	40.32±0.80	**0.002**
	**Pregnant women who had a previous cesarean delivery**		
	**Contraction (+) n=46**	**Contraction (-) n=10**	
Delivery week	36.82±0.67	38.65±0.40	**<0.001**

Data are shown as mean±standard deviation. p-values <0.05 (denoted in bold) were considered statistically significant.

**Table 3:** Delivery week based on the previous mode of delivery in the presence of contractions.

	Previous mode of birth	n	Mean±SD	p-value
Contractions present (+)	Nulliparous	20	39.5±1.2	**<0.001**
	Multiparous	32	38.8±1.3	
	Cesarean	46	36.8±0.7	

The data are presented as mean ± standard deviation. Statistically significant p-value is denoted in bold.

A total of 38 pregnant women who did not have contractions in the NST after treatment were discharged on the same day. It was determined that 46 pregnant women, who had previously given birth by cesarean section and were found to have contractions in the NST after IV iron treatment, underwent cesarean section within 24 h due to the regular contractions that did not recede in the NST and the accompanying progressive cervical effacement and dilation. The hospitalization duration for nulliparous and multiparous (previously delivered vaginally) (n=52) pregnant women with contractions detected in the NST after IV iron therapy was determined to be 2.23±0.42 days. It was observed that the contractions in the NST regressed after an average of 36.26±2.82 h.

Out of the 21 newborns requiring intensive care, 18 were infants of pregnant women who had previously delivered by cesarean section and who underwent cesarean section at a mean of 36.8±0.7 weeks due to contractions during the NST post-IV treatment (p=0.013). Two of the newborns were the infants of nulliparous pregnant women (delivery week 39.5±1.2) and one of the newborns was the infant of multiparous pregnant women (delivery week 38.8±1.3) (p=0.270, p=0.762, respectively).

## DISCUSSION

In our clinic, we identified that 72.1% of pregnant women with anemia who were 35 weeks and above had regular contractions in their NST after IV iron treatment. The average gestational age for birth in pregnant women with contractions after IV iron treatment was 39.51±1.25 for nulliparous women, 38.83±1.32 for multiparous women, and 36.82±0.67 for women with previous cesarean section. Pregnant women with previous cesarean section were found to deliver at early term.

In iron deficiency anemia, IV iron therapy is increasingly preferred due to its rapid improvement of hemoglobin levels, fewer gastrointestinal side effects, and improved safety profile^13^. Nevertheless, adverse effects such as infusion reactions and hypophosphatemia have been reported after IV iron administration^14^. However, there are no studies in the literature reporting contractions detected in the NSTs after IV iron therapy.

Calcium and iron are divalent elements that have a close relationship inside and outside the cells. It has been shown that high extracellular calcium concentration in the intestines inhibits iron absorption^15^. However, some studies have reported that this effect is temporary and that intracellular iron uptake improves shortly thereafter^9^. There are not many studies in the literature regarding whether high iron concentrations affect the intracellular uptake of calcium. In a study conducted by Núñez et al. on neuron cells, excessive iron levels were reported to increase uncontrolled calcium flux, promoting oxidative stress, and damaging mitochondrial function, as well as cell permeability^16^. The second study in the literature on this topic is by Paterek et al., who conducted research on rat cardiac muscle cells. In their study, they administered high doses of ferric carboxymaltose to rats and found that L-type Ca^2+^ channels in cardiac muscle cells were permeable to Fe^2+^ cations, allowing iron ions passing through the channels to inhibit the Ca^2+^-dependent inactivation of L-type Ca^2+^ currents, leading to increased permeability for both Ca^2+^ and Fe^2+^. Therefore, it was observed that high iron concentrations temporarily increase calcium entry into the cell^17^. L-type calcium channels are important channels present in uterine smooth muscle cells and play a role in uterine contractions during labor^18^. Uterine L-type calcium channel activity is significantly regulated throughout pregnancy and becomes more sensitive during term (35–36 weeks)^19^. Therefore, in our study, in order to clearly demonstrate the effect of IV iron treatment on uterine muscle, which was our hypothesis, we included pregnancies of 35 weeks and above in which L-type Ca channels are more active.

In our study, we believed that the high iron concentrations resulting from IV iron treatment in pregnant women beyond 35 weeks increase calcium influx through more sensitive L-type Ca^+2^ channels in the matured uterus and initiate uterine contractions. At the same time, the regression of contractions 24–48 h after IV iron application in the NST follow-ups of both nulliparous and multiparous pregnant women and the average delivery occurring at around 39–40 weeks upon discharge support the notion that this situation is temporary. However, patients with previous cesarean section are immediately taken for a cesarean section by clinicians when regular contractions are observed in NST after IV iron treatment, due to the fear of uterine rupture risk, without waiting for the 39th week. Studies have shown that in pregnant women with previous cesarean section, there is no significant difference in maternal adverse outcomes for any gestational week at or above 36 weeks, but there is an impact on neonatal adverse outcomes^20,21^. Ma'ayeh et al. reported that birth at 37 weeks in women with repeated cesarean section was associated with reduced neonatal morbidities, respiratory distress syndrome, transient tachypnea of the newborns, and neonatal intensive care unit admissions compared to birth at 36 weeks^20^. Glavind et al. showed that even between 38 and 39 weeks of gestation, there were differences in the risk of neonatal morbidity, respiratory morbidity, and neonatal hospitalization, with lower risks at 38 weeks of gestation^21^. In our study, 85% of the newborns requiring intensive care were born to mothers who had previously given birth by cesarean section, and after IV treatment, these newborns were delivered by cesarean section at an average of 36.8±0.7 weeks due to contractions detected during NST. The presence of regular contractions on NST in women with previous cesarean section triggers concerns about the risk of uterine rupture. However, Stenson et al. reported that among 208 pregnant women with previous cesarean section, they induced labor using vaginal PGE2 (prostaglandin-E2), amniotomy, oxytocin, misoprostol, and balloon catheter, and only nine cases (4.3%) experienced uterine rupture^22^.

An important limitation of our study is the absence of other adverse neonatal outcomes (neonatal morbidities, respiratory distress syndrome, transient tachypnea of the newborn) and cases of uterine rupture, excluding the need for neonatal intensive care in our analysis. Another limitation is that pregnant women under 35 weeks of gestation were not included and it could not been shown whether IV iron therapy leads to preterm delivery in them. The strength of our study is that this is the first study in the literature to question and discuss the effect of IV iron treatment on the uterus. However, animal experiments are needed to clearly demonstrate the effects of IV iron therapy on the uterus.

In conclusion, we have shown that temporary contractions may occur in NST after IV iron replacement in pregnant women with iron deficiency anemia at or above 35 weeks of gestation. We think that clinicians experience a lot of fear of uterine rupture in pregnant women who have had previous cesarean section. It is important to prevent early term labor because of concerns about adverse neonatal outcomes.

## ETHICS APPROVAL

For studies with human subjects include the following. All procedures followed were in accordance with the ethical standards of the responsible committee on human experimentation (institutional and national) and with the Helsinki Declaration of 1975, as revised in 2008. Local Ethics Committee approval was obtained from the same hospital (Approval No: 21/1029). Informed consent was obtained from all patients for being included in the study.
